# Genome-wide analysis of MYB transcription factors of *Vaccinium corymbosum* and their positive responses to drought stress

**DOI:** 10.1186/s12864-021-07850-5

**Published:** 2021-07-22

**Authors:** Aibin Wang, Kehao Liang, Shiwen Yang, Yibo Cao, Lei Wang, Ming Zhang, Jing Zhou, Lingyun Zhang

**Affiliations:** Research & Development Center of Blueberry, Key Laboratory of Forest Silviculture and Conservation of the Ministry of Education, College of Forestry, Beijing Forestry University, 35 QinghuaEast Road, 100083 Beijing, China

**Keywords:** Blueberry, Genome-wide identification, Expression profile, MYB transcription factor, Drought stress

## Abstract

**Background:**

Blueberry (*Vaccinium corymbosum* L.) is an important species with a high content of flavonoids in fruits. As a perennial shrub, blueberry is characterized by shallow-rooted property and susceptible to drought stress. MYB transcription factor was reported to be widely involved in plant response to abiotic stresses, however, the role of MYB family in blueberry responding to drought stress remains elusive.

**Results:**

In this study, we conducted a comprehensive analysis of VcMYBs in blueberry based on the genome data under drought stress, including phylogenetic relationship, identification of differentially expressed genes (DEGs), expression profiling, conserved motifs, expression correlation and protein-protein interaction prediction, etc. The results showed that 229 non-redundant MYB sequences were identified in the blueberry genome, and divided into 23 subgroups. A total of 102 MYB DEGs with a significant response to drought stress were identified, of which 72 in leaves and 69 in roots, and 8 differential expression genes with a > 20-fold change in the level of expression. 17 DEGs had a higher expression correlation with other MYB members. The interaction partners of the key VcMYB proteins were predicted by STRING analysis and in combination with physiological and morphological observation. 10 key *VcMYB* genes such as *VcMYB8, VcMYB102* and *VcMYB228* were predicted to be probably involved in reactive oxygen species (ROS) pathway, and 7 key *VcMYB* genes (*VcMYB41*, *VcMYB88* and *VcMYB100, etc.*.) probably participated in leaf regulation under drought treatment.

**Conclusions:**

Our studies provide a new understanding of the regulation mechanism of *VcMYB* family in blueberry response to drought stress, and lay fundamental support for future studies on blueberry grown in regions with limited water supply for this crop.

**Supplementary Information:**

The online version contains supplementary material available at 10.1186/s12864-021-07850-5.

## Background

Blueberry is an important perennial shrub within the genus *Vaccinium* of the Ericaceae and its fruit is rich in anthocyanin, which has significant value to human health [1, 2]. In recent years, drought has become a major threat to crop production with the climate change [3]. Blueberries are shallow-rooted plants with slender and underdeveloped roots [4], which is therefore more vulnerable to drought stress. Across the globe, drought stress led to reduced blueberry yield by about 25–30 % [5], which has evidently become a crucial factor in limiting the blueberry supply and production chain. Nevertheless, it is still unclear about the drought-tolerance mechanisms for blueberry seedlings.

Plants are universally confronted with many extreme environmental events during its lifetime. In order to survive, plants have evolved a set of elaborate and complicated self-regulation networks. Transcription factors (TFs) usually function as key regulators in plants responding to abiotic and biotic stresses [6–8]. Among them, MYB TF family was reported to be widely involved in a range of abiotic stresses [9].

MYB TF family exists in all eukaryotes and the MYB domain is generally composed of 1 ~ 4 imperfect amino acid sequence repeats (R) with about 52 amino acids in plants. MYB TFs are classified into four subfamilies (1R-MYB, R2R3-MYB, 3R-MYB, and a typical-MYB) based on the number and position of these repeats in their DNA-binding domains [10, 11]. R2R3-MYB is the main type of TF identified in the plant up to now, with more than 130 members in *Arabidopsis* and 90 in *Oryza sativa* [12]. To date, studies on MYB TF family responding to abiotic stress have been reported in many species. A total of 196, 155 and 265 *MYB* genes were identified in *Arabidopsis*, *Oryza sativa*, and *M. truncatula*, respectively [11–13]. In *Arabidopsis*, 51 % of *AtMYB* genes are up-regulated and 41 % are down-regulated under drought condition [14]; in *Oryza sativa*, 65 % of the expressed *MYB* genes were differentially regulated by drought stress in seedlings [15]. Multiple complex biological processes are involved in MYB TFs-regulated drought tolerance in plants such as ROS signaling pathway, stomatal regulation, cell strtuctue and component regulation and phytohormones-mediated signaling pathway. For example, in *Arabidopsis*, *AtMYB44* responds to drought stress by participating in stomatal regulation and ROS accumulation [16–18]. *AtMYB15* and *AtMYB2* act as positive regulators under drought stress by activating the dehydration response genes such as *AtRD22*; overexpression of *AtMYB52* can improve drought tolerance of transgenic *Arabidopsis* by affecting the cell wall structure [19, 20]. *AtMYB96* regulated cuticular wax biosynthesis and contributed to drought resistance in *Arabidopsis* [21, 22]. In woody palnts, *MdS1MYB1* particapated in positive regulation of drought stress by activating the auxin signaling pathway in apples (*Malus* × *domestica*) [23]. In addition, MYB TFs can simultaneously respond to a variety of adverse circumstances. In soybean (*Glycine max*), *GmMYB118* maintains cell homeostasis by regulating osmotic and oxidative substances, thus improving the tolerance of soybean to drought and salt stress [24]. Overexpression of *PtsrMYB* in tobacco (*Nicotiana tabacum* L.) confers enhanced salt, dehydration and cold tolerance with lower levels of ROS in transgnic tabacco compared to wild type plants [25].

In this study, genome-wide analysis of the MYB family in blueberry under drought stress was performed. Two hundred and twenty-nine non-redundant MYB sequences were identified and divided into 23 subgroups. A total of 102 MYB DEGs responding to drought stress were identified. Furthermore, 23 important potential *VcMYBs* were determined based on the expression correlation analysis and 20-fold DEGs identification. Among them, 10 key genes were probably involved in the ROS pathway, and 7 key genes were likely to be involved in leaf regulation responding to droughts stress. The key *VcMYBs* and the possible interation proteins identified in this study provide the candidate genes for genetic improvement and lay the basis for the future research to explore the molecular mechanisms of blueberry grown in regions with limited water supply for this crop.

## Results

### Identification and classification of VcMYBs from blueberry

299 cDNA sequences were identified with full-length ORFs as putative *MYB* genes from the transcriptome database on the free online platform of Majorbio Cloud Platform (https://cloud.majorbio.com/), and all potentially redundant VcMYBs sequences were discarded by ClustalX software. We performed a BLAST search against the blueberry genome database (https://www.vaccinium. org/) to verify the validity of the 299 cDNA sequences, then designated them as *VcMYB1* to *VcMYB229* for further analysis (Additional file 1). Based on the number of repeat units at the MYB-domain, the screened *VcMYBs* were classified into four groups, namely, “1R-MYB,” “R2R3-MYB,” “3R-MYB,” and “a typical-MYB”. We found that the number of the four types of *VcMYB* genes was 19 (8.30 %), 191 (83.41 %), 10 (4.37 %), and 9 (3.93 %), respectively. The subfamily R2R3-MYB had the largest number of MYB proteins in blueberry (Table 1).

**Table 1:** The MYB-domain analysis of *VcMYB* genes based on GRAVY and molecular weight

MYB groups	NO. of genes	Length(aa)			Molecular weight(D)			PI			GRAVY		
		**Min.**	**Max.**	**Avg.**	**Min.**	**Max.**	**Avg.**	**Min.**	**Max.**	**Avg.**	**Min.**	**Max.**	**Avg.**
R1	19	82	365	234	8504.77	39945.22	26196.48	5.06	11.31	8.29	-1.173	0.339	-0.605
R2R3	191	71	1686	322	7960.18	191083.53	36312.39	4.48	10.34	7.00	-1.089	-0.251	-0.704
3R	10	165	1053	607	19138.92	116410.87	67653.75	5.05	9.07	7.23	-0.949	-0.317	-0.688
Atypical	9	125	836	343	14255.23	95148.06	38713.31	5.31	9.86	7.10	-0.887	-0.084	-0.629
All	229	71	1686	328	7960.18	191083.53	36936.06	4.48	11.31	7.12	-1.173	0.339	-0.692

The physicochemical characteristics analysis of these VcMYB proteins showed that the 229 predicted VcMYB proteins varied from 71 (VcMYB40) to 1686 (VcMYB137) amino acids in length, with an average length of 328 aa, suggesting the functional diversity for VvMYB family in blueberry. The molecular weight of these VcMYB proteins ranged from 7.96 kD for VcMYB40 to 191.08 kD for VcMYB137. The mean value of GRAVY and pI was − 0.69 and 7.12, respectively (Table 1, Additional file 1 and Additional file 2).

### Phylogenetic analysis of *VcMYB* genes

**Fig. 1:**
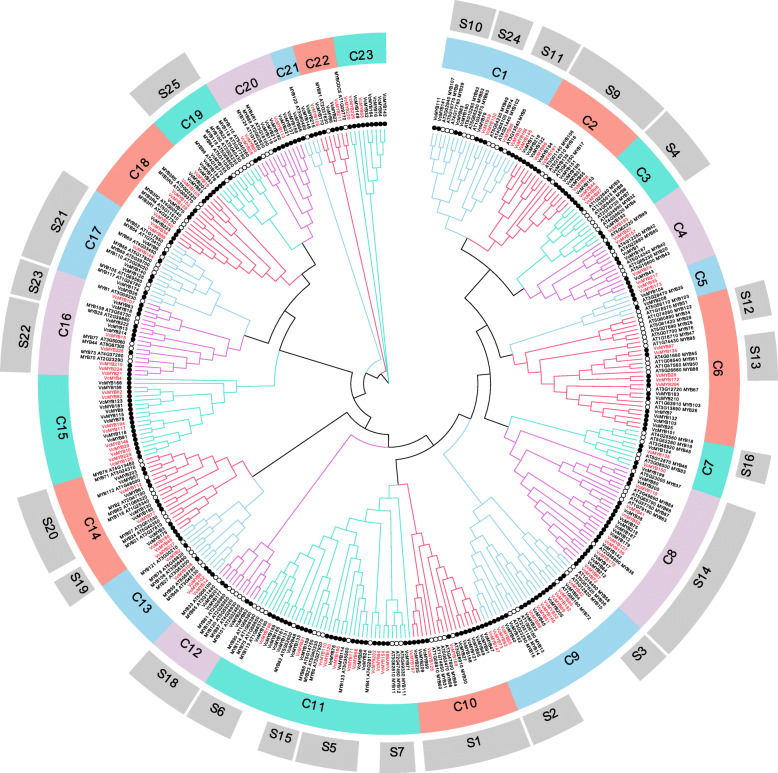
Phylogenetic analysis of MYB proteins between blueberry and *Arabidopsi*s. Complete sequence alignments of 229 MYB amino acid sequences in blueberry and 133 MYB amino acid sequences in *Arabidopsis* were performed to construct the phylogenetic tree. The black filled circle denotes *VcMYB* genes; the hollow circle denotes *AtMYB* genes. The red font indicates differentially expressed genes

In order to predict the potential function of VcMYB, we performed phylogenetic reconstruction between 229 *VcMYB* genes and 133 *AtMYB* genes using the NJ method (Fig. 1 and Additional file 1). An unrooted composite phylogenetic tree was created, in which *VcMYBs* were classified into 23 major groups (C1-C23) with supported bootstrap values. We found that subgroup C11 had the largest number of MYB proteins with 38, whereas C21 has the lowest number with only 4 members. In *Arabidopsis*, 126 *AtMYB* genes were divided into 25 subfamilies [26, 27], and these defined clades were compared and labeled with the composite evolutionary tree in our study (Fig. 1). In the evolutionary tree, 14 subgroups (C1, C2, C3, C8, C10, etc.) in our study were matched with 23 *Arabidopsis* subgroups (S1, S2, S3, S4, S5, etc.) reported in previous study [27]. However, S8 and S17 *Arabidopsis* subgroups were not retrieved from the composite evolutionary tree and no *AtMYBs* genes in *Arabidopsis* were matched with the subgroups C15 and C23 in blueberry (Fig. 1), suggesting that some divergence occurred in *MYB* family among different plant species during the long evolutionary process.

### Identification and cluster analysis of differently expressed *VcMYB* genes under drought stress

**Fig. 2:**
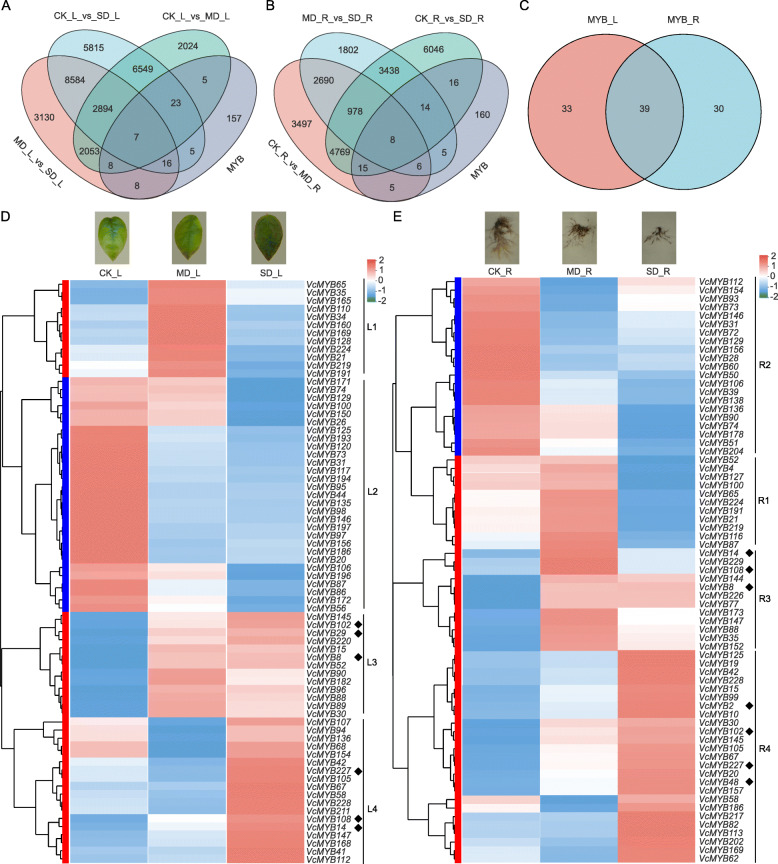
Identification and cluster analysis of differently expressed *VcMYB* genes in blueberry under drought stress. Venn diagram of DEGs in response to drought in leaves (**A**) and in roots (**B**). (**C**) represents the DEGs co-expressed in leaf and root. (**D**) and (**E**) represent the hierarchical cluster and heatmap of DEGs in leaf or root, respectively. Red indicates up-regulated genes, and blue indicates down-regulated genes. CK_L represents control in leaf; CK_R represents control in root; MD_L represents moderate drought treatment in leaf; MD_R represents moderate drought treatment in root; SD_L represents severe drought treatment in leaf; SD_R represents severe drought treatment in root; MYB_L represents the DEGs in the leaf of blueberry; MYB_R represents the DEGs in root of blueberry. The diamond indicates 20-fold *VcMYB* DEGs. L1 represents the genes with high expression of MD_L. L2 represents the genes with high expression of CK_L. L3 represents the genes with high expression of MD_L and SD_L. L4 represents the genes with high expression of SD_L. R1 represents the genes with high expression of MD_R. R2 represents the genes with high expression of CK_R. R3 represents the genes with high expression of MD_R and SD_R. R4 represents the genes with high expression of SD_R

The expression level of each gene was calculated according to the FPKM (Fragments per kb per million reads method) on the free online platform of Majorbio Cloud Platform and RSEM (http://deweylab.biostat.wisc.edu/rsem/) was used to quantify gene abundances. The differential expression analyses of *VcMYBs* were performed with the R package DESeq2 (http://bioconductor.org/ packages/stats/bioc/DESeq2/). As shown in Fig. 2, 69 and 72 differentially expressed genes (DEGs) were screened out (at a fold ratio with 2 and P-adjusted value with 0.05) in leaf and root, respectively (Fig. 2 A and B). A total of 102 DEGs were identified in leaves and roots, among which 39 DEGs were found to be co-expressed both in leaves and roots, whereas 33 and 30 genes specially expressed in leaves and roots, respectively (Fig. 2 C).

In order to facilitate the analysis of these DEGs, a heatmap was constructed based on log_10_ FPKM (Fig. 2D and E). Under drought stress, the expression of DEGs in leaves and roots can be clustered into 2 groups, namely the up-regulated group (L1, L3, L4, R1, R3 and R4) and the down-regulated group (L2 and R2). The number of up-regulated DEGs was 43 in leaves and 48 in roots, and down-regulated DEGs in leaves and roots was 29 and 21, respectively. Meanwhile, eight 20-fold differential genes were identified, and clustered into C13 (*VcMYB2*, *VcMYB8*, *VcMYB14*, *VcMYB29*, *VcMYB48*, *VcMYB102*, *VcMYB108* and *VcMYB227*) and C15 (*VcMYB29*) subfamilies (Fig. 1).

In order to verify the accuracy of RNA-seq data and the reliability of the data analysis, we randomly selected 16 *VcMYB* TFs for qRT-PCR analysis in leaves and roots. We found that the qRT-PCR results were basically consistent with the RNA-seq data, except that the expression level of *VcMYB2* in leaves showed slight difference. Our results indicated that the RNA-Seq data in this study were accurate and reliable (Additional file 3).

### Phylogenetic and conserved motifs analysis of *VcMYB* DEGs

**Fig. 3:**
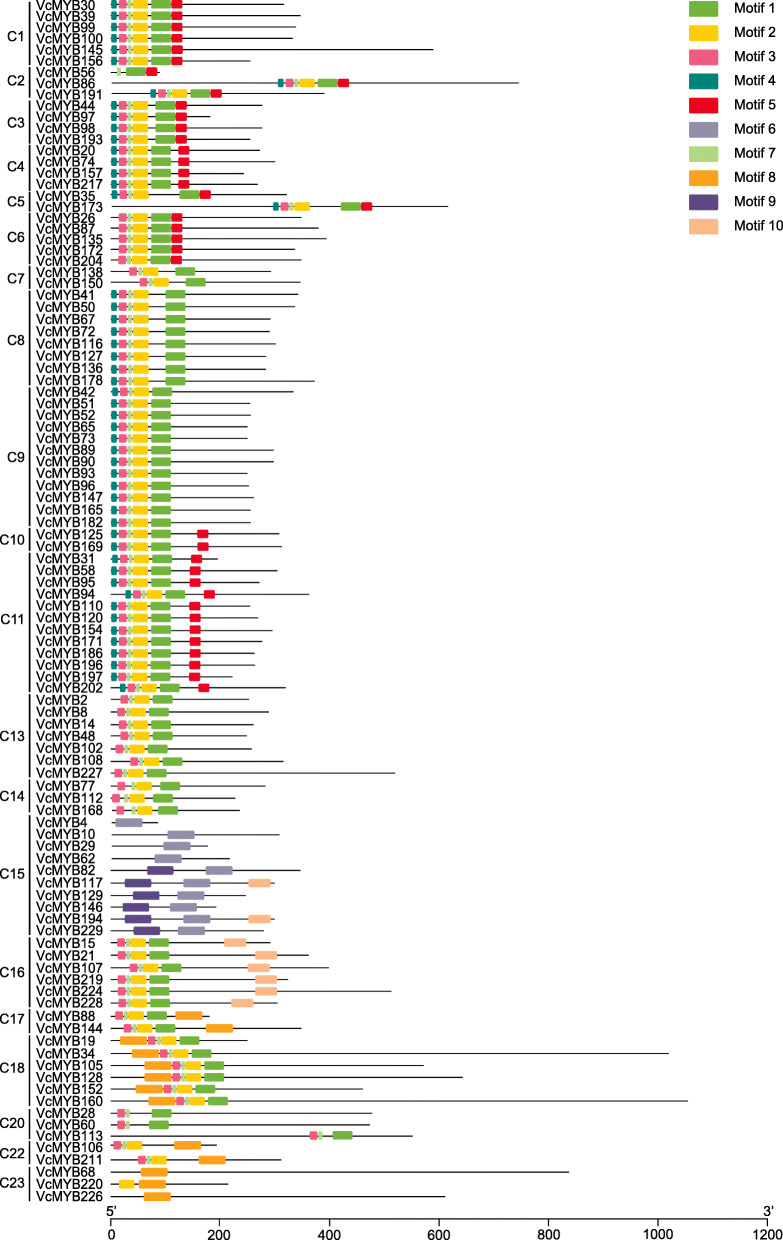
Phylogenetic tree and conserved motifs analysis of *VcMYB* DEGs. The phylogenetic tree was constructed using MEGA6.0 software, and was decorated by TBtools software. MEME (v4.11.1) online program was employed to predict the motif

In order to further analyze the characteristics of these 102 DEGs in 23 subgroups, the conserved motifs of VcMYB proteins were analyzed using the MEME online program. 10 motifs, named motif 1–10 (Additional file 4) were determined for these MYB DEGs proteins (Fig. 3), among which motif 1, 6 and 7 were identified as the core conserved domains and constituted the SANT domain of MYB (Additional file 4). Among these conserved motifs, motif 3 and 7 correspond to the most conserved genes with 88, and motif 9 corresponds to the least conserved genes with only 6 based on the prediction. Motif 6 and 8 contained 50 amino acids, but motif 7 contained only 8 amino acids (Additional file 4). It should be noted that no differential genes were identified in C12, C19 and C21 subfamilies. VcMYB proteins in the same cluster subgroup were observed to have the similar motif composition (Fig. 3). For example, subgroup C20 contained motif 1, 3 and 7, indicating the functional similarities in the same subgroup [28, 29].

**Fig. 4:**
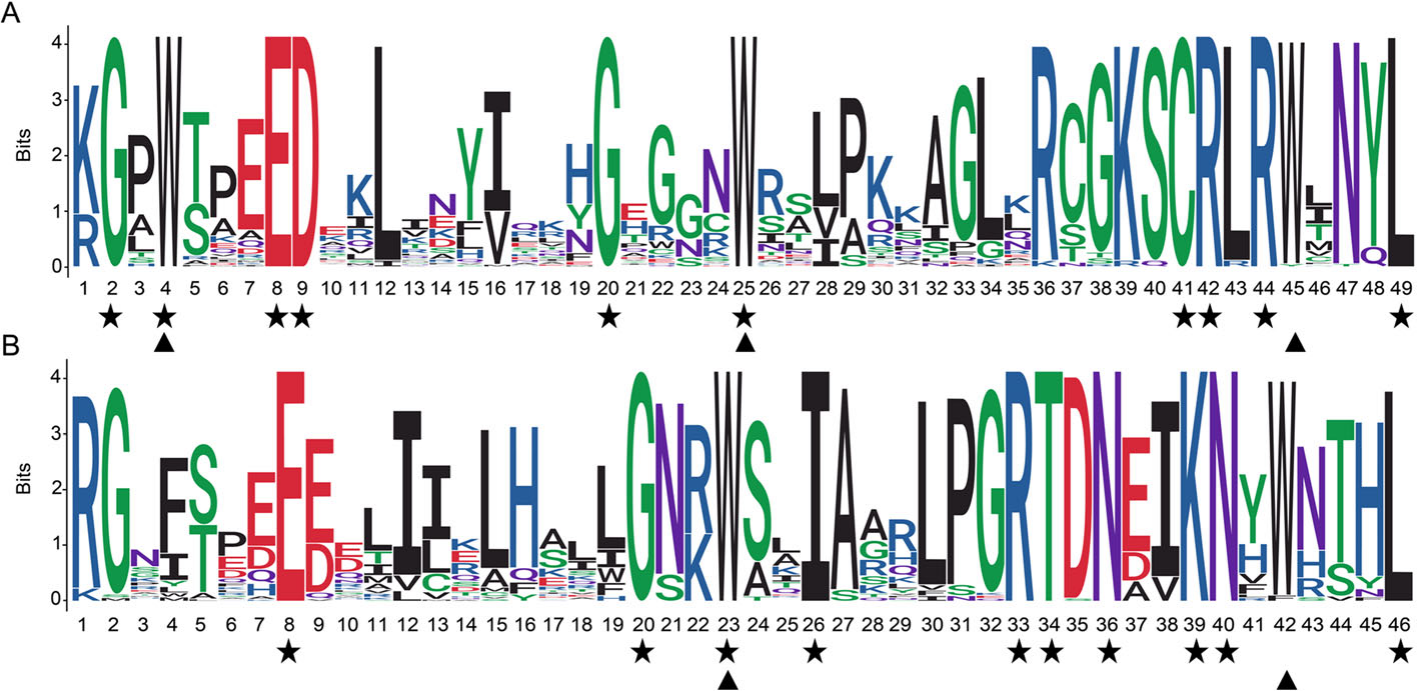
The sequence logos of the R2 (**A**) and R3 (**B**) VcMYB repeats. These logos were based on multiple full-length alignments of all blueberry R2R3-MYB domains. The bit score represents the information content for each position in the sequence. Asterisks represent the conserved residues that are identical among all R2R3-MYB domains, and triangles denote the typically conserved residues (Trp) in the R2R3-MYB domains. Gly (G), Glu (E), Asp (D), Cys (C), Arg (R), Leu (L), Iie (I), Thr (T), Asn (N) and Lys (K)

In order to elucidate the sequence feature of these VcR2R3-MYB proteins in blueberry, multiple sequence alignment was perfomed by using Clustal X. 71 highly conserved VcR2R3-MYB amino acid regions were identified among 102 differently expressed VcMYB proteins (such as VcMYB2, VcMYB8, VcMYB14 etc.) (Additional file 5). Subsequently, ggseqlogo was used to generate sequence logos. The R2 and R3 MYB repeats of the VcR2R3-MYBs contained many conserved amino acids, such as the typical tryptophan (Trp), which is crucial to the sequence-specific binding of DNA[28]. Five conserved Trp residues were identified in the R2 and R3 repeats (Fig. 4 A and 4B). As with its counterparts in the Chinese Pear (*Pyrus bretschneideri* Rehd.) [28], the first conserved Trp residue in the R3 repeat was generally replaced by other amino acid (Fig. 4B), meanwhile, the second and third conserved Trp residue were identified in the R3 repeat in bluebrerry. Some other highly conserved amino acids were also discovered, such as Gly (G), Glu (E), Asp (D), Cys (C), Arg (R), Leu (L), Iie (I), Thr (T), Asn (N) and Lys (K).

### GO annotation analysis of *VcMYB* DEGs

The potential functions of DEGs for *VcMYB* in blueberry were predicted by GO annotation analysis. In this study, the 102 *VcMYB* DEGs were assigned to 16 GO categories (Additional file 6 and Additional file 7). The results showed that 102 DEGs participated in the biological process (38), cellular component (7) and molecular function (21). The “metabolic process” gene (11) was the dominant category in the biological process category accounting for 16.92 %. *VcMYB48*, *VcMYB88*, *VcMYB108*, *VcMYB229*, *VcMYB129* and *VcMYB228* were categorized into responding to stimulus. In the cellular component category, *VcMYB* genes were mainly located in the “cell,” “organelle,” and “cell part.” Regarding the molecular function category, “binding” (14) was the most dominant group accounting for 21.54 % (Additional file 6).

**Fig. 5:**
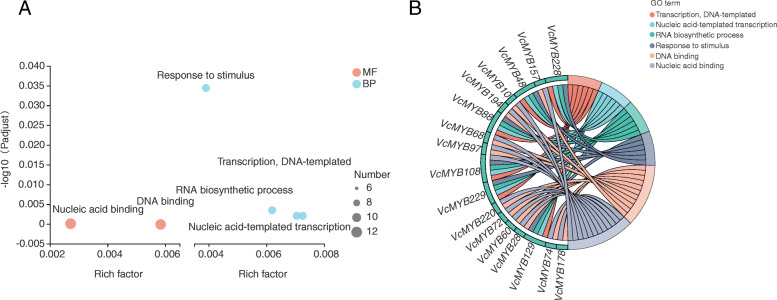
Gene ontology enrichment analysis of DEGs. (**A**) Bubble diagram of DEGs based on GO functional-enrichment analysis. MF represents molecular function, and BP represents biological process. (**B**) String diagram of DEGs on the basis of important GO terms

GO functional enrichment analysis was further carried out by Goatools based on the Majorbio Cloud Platform. A total of 6 GO terms were considered to be significantly enriched among these DEGs, in which 4 and 2 GO terms belonged to “biological process” (BP) and “molecular function” (MF), respectively (Fig. 5 A). By analyzing the genes involved in the 6 GO terms, we found that *VcMYB108* participated in the most of GO terms (5), and *VcMYB228*, *VcMYB48*, *VcMYB88*, *VcMYB229* and *VcMYB129* participated in 4 GO terms, respectively (Fig. 5B).

### Expression correlation analysis of *VcMYB* DEGs in blueberry

**Fig. 6:**
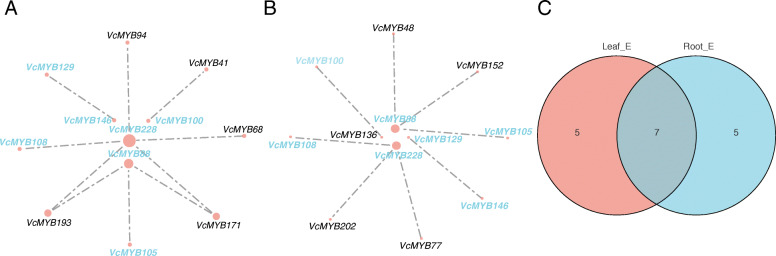
Expression correlation analysis of DEGs in blueberry under drought stress. DEGs’ expression correlation network under drought stress in leaf (**A**) and in root (**B**). Lines represent expression correlation between genes, and each node represents an MYB gene. Large nodes represent that this gene has an expression correlation with a large number of other genes. The blue font indicates that this gene coexists in the expression correlation network of the leaf and the root. The black font indicates that this gene is specifically present in the expression correlation network of the leaf or the root. (**C**) Venn diagram of DEGs that were expressed in leaf or root

The possible co-expression relationship between genes can be reflected by the correlation analysis of expression levels of genes. Therefore, we constructed two expression correlation networks of 72 DEGs in leaf and 69 DEGs in root in blueberry based on the spearman correlation algorithm. The results showed that 12 *VcMYB* genes were correlated in the expression correlation network of leaves and roots (Fig. 6 A and 6B), among which 7 genes were co-expressed including *VcMYB88*, *VcMYB100*, *VcMYB105*, *VcMYB108*, *VcMYB129*, *VcMYB146* and *VcMYB228* (Fig. 6 C). Notably, *VcMYB88* and *VcMYB228* had the largest node whether in root or leaf, indicating that the two genes were significantly correlated with other genes and may play a crucial role in blueberry response to drought stress.

### 10 *VcMYB* DEGs probably participated in drought response through ROS-mediated signaling pathway

**Fig. 7:**
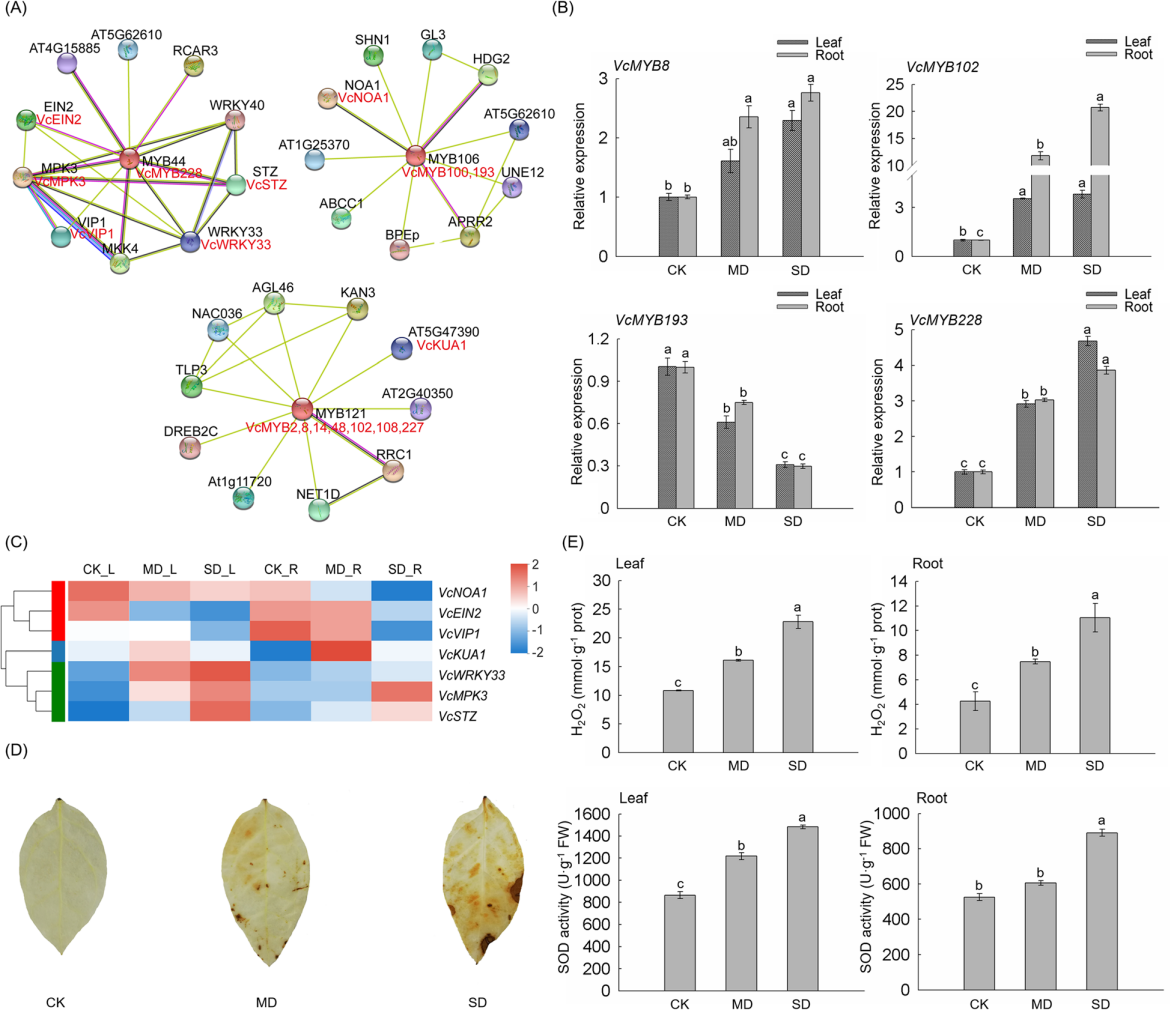
Effect of drought stress on SOD and H_2_O_2_ of blueberry. (**A**) Protein-protein interaction analysis of 10 *VcMYB* DEGs by STRING. (**B**) The expression profile of *VcMYB8*, *VcMYB102*, *VcMYB193* and *VcMYB288* using qRT-PCR under drought stress. Each value is represented as the mean value ± standard error of three independent determinations, and different letters indicate significant differences at *p* < 0.05 by Duncan’s multiple range test. CK: control group; MD: moderate drought stress; SD: severe drought stress. (**C**) The expression heatmap of the interacting proteins in leaf and root. (**D**) In situ visualization of H_2_O_2_ accumulation by DAB staining of the blueberry leaf under drought stress. (**E**) SOD activity and H_2_O_2_ content in leaf or root. Each value is indicated as the mean value ± standard error of three independent determinations, and different letters represent significant differences at *p* < 0.05 by Duncan’s multiple range tests

According to the above screening, eight 20-fold differential genes including *VcMYB2*, *VcMYB8*, *VcMYB14*, *VcMYB48*, *VcMYB29*, *VcMYB102*, *VcMYB108* and *VcMYB207* were identified (Fig. 2). The expression correlation analysis of MYB TFs showed that 17 genes (Fig. 6) were significantly correlated in the expression correlation network of blueberry leaves and roots. Thus, based on the orthologs of *Arabidopsis*, we further predicted the potential interaction proteins of these 10 key VcMYB proteins by STRING analysis to explore the possible functioin of VcMYB protein in blueberry response to drought stress (Fig. 7 A). The results showed that multiple candidate key proteins interact with each other, i.e., MYB44 (homolog of VcMYB228) could interact with EIN2, MPK3, VIP1, WRKY33 and STZ (homolog of VcEIN2, VcMPK3, VcVIP1, VcWRKY33 and VcSTZ, respectively), MYB106 (homolog of VcMYB100 and VcMYB193) could interact with NOA1 (homolog of VcNOA1), MYB121 (homolog of VcMYB2, VcMYB8, VcMYB14, VcMYB48, VcMYB102, VcMYB108 and VcMYB227) could interact with AT5G47390 (homolog of VcKUA1) (Fig. 7 A and Additional file 8). Previous studies reported that overexpression of *AtMYB44*, *AtEIN2*, *AtMPK3*, *AtVIP1*, *AtWRKY33*, *AtSTZ*, *AtMYB106*, *AtNOA1*, *AtMYB121* and *AtKUA1* could enhance drought tolerance by regulating the accumulation of ROS and cell homeostasis [16, 18, 30–33]. Therefore, we speculate that the 10 key *VcMYB* DEGs were likely to be involved in drought response through ROS-mediated signaling pathway in blueberry.

The qRT-PCR results showed that the relative expression level of *VcMYB8*, *VcMYB102* and *VcMYB228* increased significantly and *VcMYB193* decreased significantly with the duration of drought stress in leaves and roots (Fig. 7B). Furthermore, the relative expression levels of the other 6 potentially key *VcMYB*s (*VcMYB2*, *VcMYB14*, *VcMYB48*, *VcMYB100*, *VcMYB108* and *VcMYB227*) were also significantly induced under drought stress in leaves and roots (Additional file 3), implying the functional divergence and significant role of these *VcMYBs* genes in blueberry under drought stress. Under drought stress, among the DEGs proteins that could interact with key VcMYBs in blueberry, VcMPK3, VcWRKY33 and VcSTZ showed trends upward, VcNOA1, VcEIN2 and VcVIP1 showed the downward trend (Fig. 7 C).

Furthermore, we examined the change of superoxide dismutase (SOD) activity and the content of H_2_O_2_ in the leaf and root of blueberry under drought stress. The results indicated that with the duration of drought stress, the content of H_2_O_2_ in leaves and roots were significantly increased, and more and more reddish-brown plaques were observed in leaves (Fig. 7Dand E), indicating that drought stress increased the amount of oxygen released by H_2_O_2_. Nevertheless, the activity of SOD in leaves and roots increased significantly with the aggravation of drought treatment (Fig. 7E).

### 7 *VcMYB* DEGs probably participated in drought response through the leaf regulation pathway

**Fig. 8:**
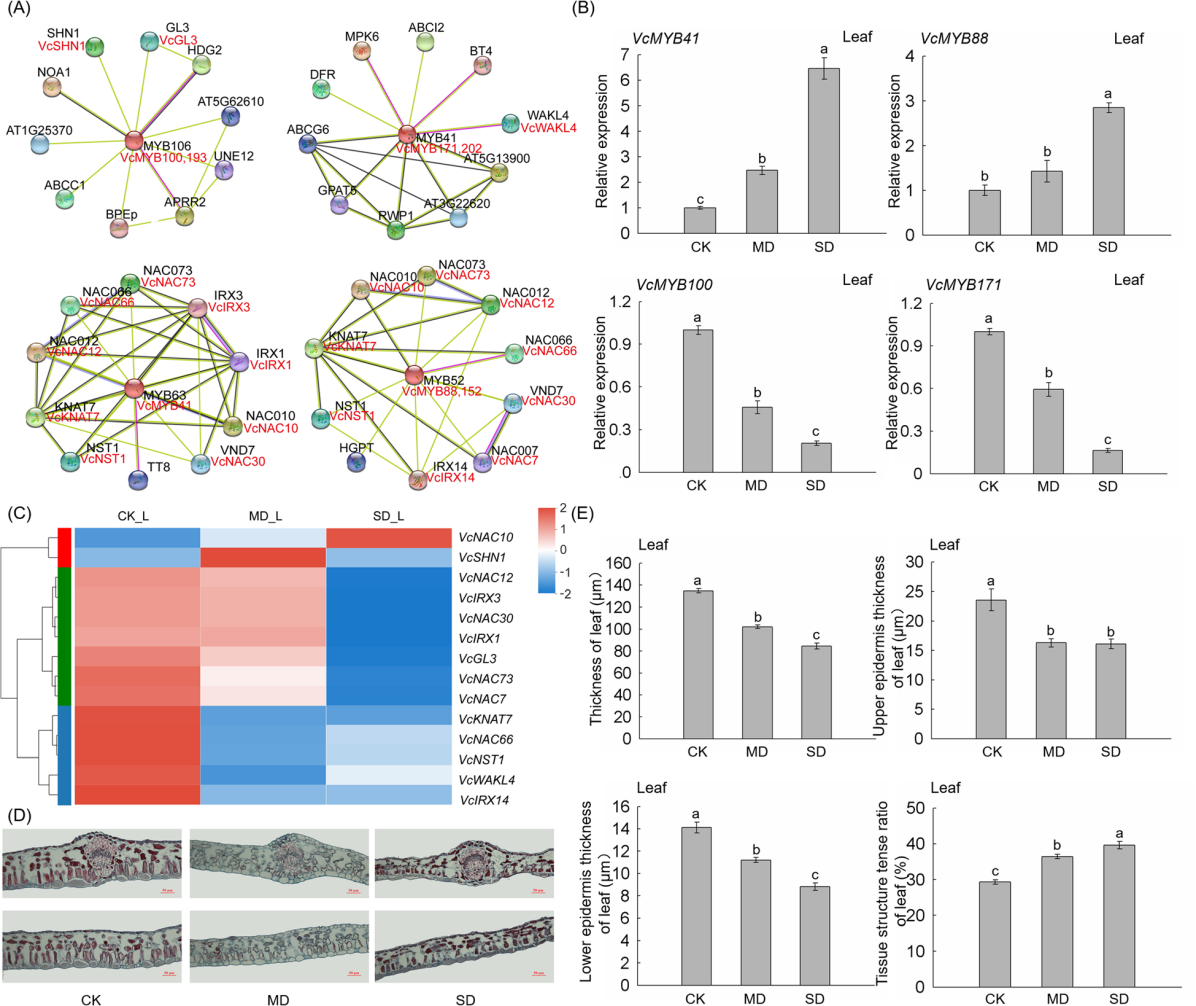
Morpho-anatomical trait and structural parameter changes of blueberry under drought stress. (**A**) Protein-protein interaction analysis of 7 *VcMYB* DEGs by STRING. (**B**) The expression profile of *VcMYB41*, *VcMYB88*, *VcMYB100* and *VcMYB171* under drought stress. Each value is represented as the mean value ± standard error of three independent determinations, and different letters indicate significant differences at *p* < 0.05 by Duncan’s multiple range test. (**C**) The expression heatmap of interacting proteins in leaf. (**D**) Morpho-anatomical trait changes of blueberry under drought stress. (**E**) Total thickness, upper epidermis thickness, lower epidermis thickness and tissue structure tense ratio of the leaf. Each value is indicated as the mean value ± standard error of three independent determinations. Different letters represent significant differences at *p* < 0.05 by Duncan’s multiple range test

In this study, based on the ortholog*s* analysis of *Arabidopsis*, we predicted the interactions of 7 key VcMYB proteins (VcMYB41, VcMYB88, VcMYB100, VcMYB152, VcMYB171, VcMYB193 and VcMYB202) of blueberry by STRING analysis (Fig. 8 A). The results showed that many candidate proteins interact with each other, for example, MYB41 (homolog of VcMYB171 and VcMYB202) could interact with WAKL4 (homolog of VcWAKL4), MYB106 (homolog of VcMYB100 and VcMYB193) could interact with SHN1 and GL3 (homolog of VcSHN1 and VcGL3, respectively), and MYB63 (homolog of VcMYB41) could interact with NAC073, IRX3, IRX1, NAC010, VND7, NST1, KNAT7, NAC012 and NAC066 (homolog of VcNAC73, VcIRX3, VcIRX1, VcNAC10, VcNAC30, VcNST1, VcKNAT7, VcNAC12 and VcNAC66, respectively) (Fig. 8 A and Additional file 8). Previous reports showed that overexpression of the interacted proteins, such as AtMYB106, AtSHN1, AtGL3, AtMYB41, AtWALK4, AtMYB63 enhanced the drought tolerance of *Arabidopsis* by affecting leaf morphology[34], cell wall biosynthesis [19, 34, 35], wax biosynthesis [36, 37] and cuticle biosynthesis [38]. The leaf morphology and structure changes occurred due to the damaged biosynthesis of leaf cell wall, wax, and cuticle [39]. Thus, we speculated that the 7 key *VcMYB* DEGs might participate in drought response through the regulation of leaf morphology and structure in blueberry.

The qRT-PCR results showed that the relative expression level of *VcMYB41*, *VcMYB88*, *VcMYB100* and *VcMYB171* in leaves changed significantly with the duration of drought stress (Fig. 8B). Meanwhile, the relative expression levels of the other 3 *VcMYB*s (*VcMYB152*, *VcMYB193* and *VcMYB202*) of the regulatory genes potentially involved in leaf morphology and structure were also significantly induced under drought stress in leaves (Fig. 7B, Additional file 3). The expression heatmap of these interaction proteins showed that these genes could respond to drought stress (Fig. 8 C), implying their possible role of these genes in response to drought stress. Moreover, we observed that the blueberry leaf cells shortened, shrunk and damaged under drought treatments (Fig. 8D). The total thickness of leaves, the thickness of the upper epidermis and lower epidermis of leaves decreased with drought stress. The tissue structure tense ratio of the leaf (CTR) showed an obvious increase with the aggravation of drought degrees. Under SD treatment, the CTR increased by 35.31 and 8 % compared to CK and MD treatment, respectively (Fig. 8E).

## Discussion

In this study, 229 MYB TFs were identified in blueberry genome and on the free online platform of Majorbio Cloud Platform. Our previous study showed that blueberry genome was closely related to *Vitis vinifera* L. [40]. In order to reveal the divergence and differences of *MYB* gene family between different plant species, we compared the numbers of MYBs from *Vitis vinifera* L. (134 *MYBs*) [41], *Pyrus bretschneideri* Rehd. (129 *MYBs*) [28], *Arabidopsis* (197 *MYBs*) [15], *Brassica rapa ssp. pekinensis* (104 *MYBs*) [42], and *O. sativa* (155 *MYBs*) [15] with the number of *VcMYBs* in blueberry. It was found that the number of *MYB* genes in blueberry was obviously higher than that in other plant species, indicating that the large number of *VcMYB* genes in blueberry, which is tetraploid, was probably realted to the occurrence of multiple whole-genome duplication (WGD) events during the species evolution. It was also reported that the divergence of subfamily or gene number in same gene family of different species is related to their genome size or evolutionary differences [43]. Interestingly, we found that C15 and C23 subgroups belonged to two newly expanded subfamilies in the phylogenetic tree (Fig. 1), which may have important significance for blueberry.

Generally, there is a quite conserved DNA domain (MYB domain) at the N-terminus of MYB protein. The domain usually includes 1–4 incomplete repeats (R), and each R domain contains 3 α-helix structures [44, 45]. Six conserved tryptophan residues were evenly distributed in the R2 and R3 domains of MYB proteins [15, 46]. In this study, we found five conserved Trp residues of the VcR2R3-MYBs conservative domain in blueberry and the first Trp residue of R3 repeats was variable. Actually, a similar phenomenon was also reported in other species, the first Trp residue of R3 repeats was also variable in *Alfalfa* [11]. Substitution at the first Trp residue may be responsible for recognizing novel target genes or may lead to loss of DNA-binding activity against target genes [28]. Compared to the relatively conserved N terminal, the C-terminal domain of MYB protein has high variability [26, 47]. In the present study, by using the MEME program, we found that the conserved motifs located at the C-terminal of VcMYB proteins were quite different, such as *VcMYB4*, *VcMYB113* and *VcMYB226*. Therefore, variable C-terminal domains and relatively conserved N-terminal domains formed the MYB gene family with functional diversity.

The expression correlation is the embodiment of the correlation coefficient between genes obtained by the spearman correlation algorithm, and is also a useful analysis method for predicting important putative genes [48]. Based on these methods, we identified 17 highly expressive genes in blueberry under drought stress. In particular, *VcMYB88* and *VcMYB228*, which had the largest node, have more correlation with other *MYB* genes, probably playing an important role in blueberry response to drought stress. In ramie (*Boehmeria nivea*), *Bn4CL3* was predicted to be involved in the synthesis of lignin through the expression correlation, and further research verified that *Bn4CL3* negatively regulates lignin synthesis in the experiment of recombinant protein [49].

Although the genome sequencing of blueberry has been completed, the functional annotation of many genes was relatively imperfect [50, 51]. Therefore, it is a useful supplementary solution to perform systematic evolution analysis in order to understand and predict the potential functions of the key genes [52]. In the differential gene analysis, we identified 8 *VcMYB* genes with 20-fold differences, and these genes were mainly classified into C13 subfamily. Furthermore, in the expression correlation analysis, 17 *VcMYB* genes with higher correlation were obtained, which were mainly classified into the C11 and C18 subfamilies. Notably, the three subgroups C11, C13 and C18 contained many *Arabidopsis MYB* genes, such as *AtMYB12*, *AtMYB41* and *AtMYB108*, and these genes have been reported to participate in plant responses to drought stress [20, 53, 54]. Therefore, we speculated that the *VcMYB* genes belonged to C18, C11and C13 subgroups, such as*VcMYB2*, *VcMYB48* and *VcMYB108*, etc., probably play crucial roles in blueberry responding to drought stress. By using qRT-PCR analysis, we confirmed that these genes could be induced under drought stress.

**Fig. 9:**
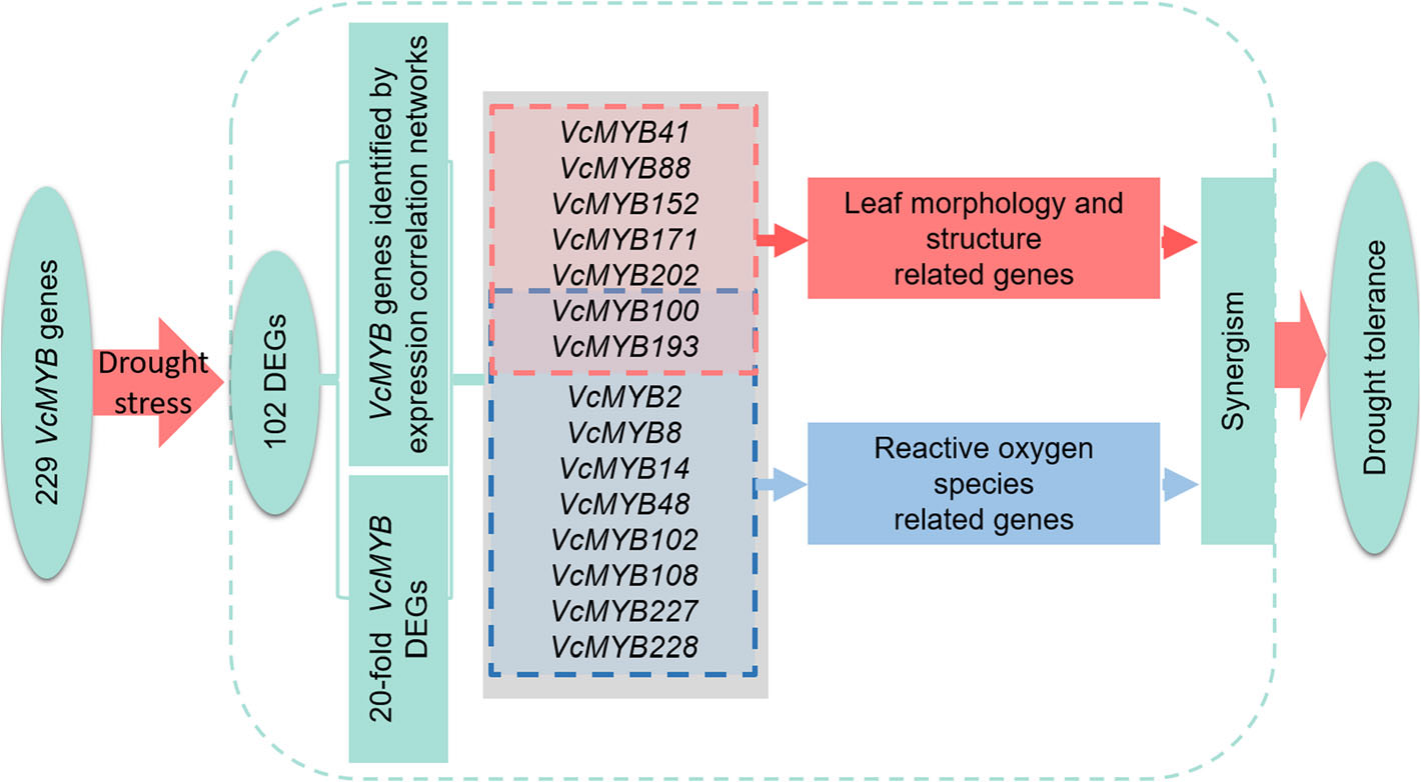
A prediction model of the mechanism of blueberry VcMYB genes in response to drought.

A body of evidence demonstrated that MYB TFs participate in drought response by regulating the ROS pathway. For example, in *Arabidopsis*, overexpression of *TaMYB33* gene improves tolerance to drought stress through the ROS pathway [55]; the *GbMYB5* overexpression in tobacco increased the tolerance to drought stress by elevating the antioxidant enzyme activities and inducing the expression of stress-responsive genes (SOD, CAT and GST) [56]. According to this study, a total of 23 key *VcMYB* genes were identified (Figs. 2 and 6). Based on the orthologs analysis of *Arabidopsis*, the interaction partners of the key VcMYB proteins were predicted by STRING analysis. 7 interaction proteins of 10 key VcMYB proteins were identified (Fig. 7) and its orthologs in *Arabidopsis* such as AtEIN2, AtMPK3, AtKNAT7, AtSHN1 and AtSTZ, etc. [31, 33, 57] were reported to function through the ROS-mediated signaling and leaf regulation pathway under drought stress. Thus, we speculated that as the interaction partners of these proteins, VcMYB228, VcMYB100, VcMYB193, VcMYB2, VcMYB8, VcMYB14, VcMYB48, VcMYB102, VcMYB108 and VcMYB227, probably play crucial roles in ROS accumulation and elimination under drought stress (Figs. 7 and 9). Most abiotic stresses can cause oxidative stress and changes of ROS in plants [58, 59]. In the present study, we also observed that the reddish-brown patches of blueberry leaves by diaminobenzidine (DAB) staining increased under drought stress, indicating that drought stress increased the amount of oxygen released by the H_2_O_2_ in cells of blueberry leaves, which in turn further increase the degree of oxidation in cells (Fig. 7 D and E). With the aggravation of drought treatment, the activity of SOD in leaves and roots increased significantly (Fig. 7E). In fact, similar results have been reported in other species. For instance, in *Manihotesculenta*, the SOD activity of leaves increased significantly under drought stress [60]. In *Brassica campestris*, SOD activity and the content of H_2_O_2_ increased obviously when plants were subjected to drought stress [61]. Nevertheless, further study are needed for the 10 key *VcMYB* DEGs we screened to reveal the accurate signaling pathways of its involvement.

The leaf is indispensable for regulating plant water transport and the changes of leaf morphology and structure is crucial for plants responding to drought stress [62]. CTR and leaf thickness are key indicators for the leaf morphology and structure [63, 64]. The leaf morphology and structure changes usually occurred due to the damaged biosynthesis of leaf cell walls, wax, and cuticle [39]. Here, we found that with the degree of drought increased, the leaf thickness of blueberries seriously decreased, while the CTR increased (Fig. 8D and E), which may be due to the disruption of xylem water supply and lead to the retardation of cell elongation and expansion [65]. In *Quercus ilex*, the leaves tended to get smaller under drought stress, while rehydration promotes larger leaves [66]. Previous studies showed that MYB TFs participates in drought response by leaf morphology and structure pathway. In *Arabidopsis*, the AtMYB96 gene overexpression improves tolerance to drought stress by changing leaf morphology [67]. In this study, based on the ortholog*s* analysis in *Arabidopsis*, the interactions of key VcMYB proteins were predicted by STRING analysis. 14 interaction proteins of 7 key VcMYB proteins were identified and its orthologs in *Arabidopsis* such as AtKNAT7, AtSHN1 and AtNST1, etc. [68–70] were reported to function through the leaf regulation pathway under drought stress. Therefore, we speculated that as the important interaction partners of these proteins, VcMYB41, VcMYB88, VcMYB100, VcMYB152, VcMYB171, VcMYB193 and VcMYB202 are probably involved in the blueberry response to drought stress by regulating the leaf morphology and structure (Figs. 8 and 9).

In order to verify the accuracy of RNA-seq data, we randomly selected 16 *VcMYB* DEGs of blueberry under drought stress, and analyzed the RNA-seq and qRT-PCR data of these genes. The results showed that the expression trend of the selected gene were basically similar for RNA-seq and qRT-PCR data, suggesting the transcriptome sequencing was accurate and validity. Nevertheless, we also found that the folds of the expression changes were different. We speculated that two different sequencing methods probably lead to a slight difference. Zhu et al. [71] found that there were some differences between the two sequencing methods under different operating conditions. Interestingly, we found that some genes in blueberries have different expression trends in leaves and roots under drought treatment. For example, the expression levels of *VcMYB20, VcMYB157* and *VcMYB226* in leaves were significantly down-regulated under drought stress, while up-regulated in roots. Meanwhile, some tissue-specific genes, such as *VcMYB48*, were not expressed in leaves, but roots, indicating that *VcMYB48* was a root-specific gene. Our study provide new promising candidates and insights for blueberry in response to drought stress.

## Conclusions

229 non-redundant *VcMYB* sequences were identified in blueberry and can be divided into 23 subfamilies. 102 DEGs that significantly responded to drought treatment were identified, including 72 in leaves and 69 in roots, and 8 differential expression genes with a > 20-fold change in the level of expression. Among 102 DEGs, 17 DEGs had higher expression correlation with other MYB members. In addition, 15 key *VcMYB* genes including *VcMYB8*, *VcMYB14*, *VcMYB88*, *VcMYB102*, *VcMYB108* and *VcMYB228 etc.*., which were probably involved in ROS and leaf morphology and structure pathways under drought treatment, were identified by STRING analysis combined with physiological and morphological observation. These findings provide new candidates and a new understanding of the regulatory mechanism of *VcMYB* genes family in blueberry response to drought stress.

## Methods

### Plant materials and experimental treatments

In this study, tissue-culture seedlings of blueberry variety ‘Bluecrop’ with a height of about 18 cm were used as the test materials. These blueberry seedlings were purchased from Dalian Senmao Modern Agriculture Co., Ltd., Dalian, China. They are very common fruit trees in China and are not endangered species. No specific permits were required for the sample collection.

In March 2018, the 3-month-old uniform tissue culture seedlings were transplanted into small plastic pots with the size of 15 × 15 cm at the top and 10 × 10 cm at the bottom. The nutrient soil included natural peat-soil and vermiculite and the volume ratio of peat-soil to vermiculite was 1:1. The available nutrients in the soil contained nitrogen with 140 mg·kg^-1^, phosphorus with 100 mg·kg^-1^, potassium with 180 mg·kg^-1^, soil organic matte with 45 % and pH value was 5.5. The seedlings were grown in the greenhouse with 16-h light/8-h dark photoperiod and watered with tap water modulated to pH 5.2–5.5 by dilute sulphuric acid. The greenhouse temperature was 25 ± 1℃ and the relative humidity was 55–65 %. All pots were irrigated with an equal amount of water every 3 days until the drought treatment was applied by withholding irrigation. The experiment was designed as a complete randomized block with three replicates per block, and nine pots were employed in each replicate. Three uniform plants were grown in each pot and three treatments were desighed as follows: CK, control group with soil water content 75–80 %; MD, moderate drought group with soil water content 55–60 %; SD, severe drought group with soil water content 30–35 %. Under the condition of greenhouse, three treatments of plants were applied by withholding irrigation 15 days after transplanting. The soil water content (SWC) reached the predetermined levels without water for 10, 20 and 40 days, respectively[7]. The gravimetric method was employed to control the SWC [72]. Every pot was weighed at 15:00 Beijing Standard Time (BST) each day. For RNA transcriptome sequencing, samples were taken when the extent of drought treatment reached the three predetermined levels, respectively. For physiological index detection, another 10-day treatment were conducted for each drought treatment after the three predetermined levels reached and then sampled. The pots were destroyed between 15:00 and 17:00 BST.

For sampling, the treated blueberry seedlings were cut off at soil level and leaves (L) and roots (R) were washed with distilled water and then wiped dry with paper towel. All samples were immediately frozen in liquid nitrogen and stored at -80℃ for further analysis. The experimental materials used for RNA-seq and qRT-PCR were single plant sample with three biological replicates. The materials used for physiological and biochemical analysis were mixed samples, and six plants were chosen as one biological replicate, and three biological replicates were included for each assay.

### Identification of the *MYB* gene family in blueberry

Samples of the three treatments were sent to Shanghai Majorbio Bio-pharm Biotechnology Co., Ltd. (Shanghai, China) for library construction and sequenced by means of Illumina HiSeq xten system (www.majorbio.com) [7]. Total RNA was extracted from the sample using TRIzol® Reagent (Plant RNA Purification Reagent for plant tissue) according the manufacturer’s instructions (Invitrogen) and genomic DNA was removed using DNase I (TaKara). RNA-seq transcriptome library was prepared following the TruSeqTM RNA sample preparation Kit from Illumina (San Diego, CA) using 5 µg of total RNA. After quantified by TBS380, the paired-end RNA-seq sequencing library was sequenced with the Illumina HiSeq xten (PE150). The raw reads were trimmed and quality controlled by SeqPrep (https://github.com/jstjohn/SeqPrep ) and Sickle (https://github.com/najoshi/sickle ) with default parameters. Then clean reads were separately aligned to a reference genome using Hisat2 with default parameters (Http://tophat.cbcb.umd.edu/,version2.0.0) software. The original data were stored in the free online platform of Majorbio Cloud Platform (https://cloud.majorbio.com/) and the National Genomics Data Center, China National Center for Bioinformation / Beijing Institute of Genomics, Chinese Academy of Sciences (https://bigd.big.ac.cn/gsub/) (CRA003258).

The sequences of VcMYB protein sequences were obtained and analyzed from the free online platform of Majorbio Cloud Platform (https://cloud.majorbio.com/) and the blueberry genome (https://www.vaccinium.org/), and the sequences of AtMYB protein sequences in *Arabidopsis* were downloaded from the *Arabidopsis* genome TAIR database (https://www.arabidopsis.org/). Subsequently, all candidate VcMYBs were verified using SMART3 and Pfam Program (http://pfam.xfam.org/) to confirm that they contained the core domains [28]. Moreover, the remaining sequences were analyzed by ClustalX software, and all potentially redundant VcMYBs sequences were discarded. Non-redundant sequences were renamed as predicted *VcMYB* genes. The theoretical isoelectric point (PI), molecular protein weight (kDa) and an average of hydropathicity (GRAVY) index values of each VcMYB were obtained using the ProtParam tool (https://web.expasy.org/protparam/) [73].

### Conserved motif and phylogenetic analysis of *VcMYB* genes

Multiple sequence alignments of the identified MYB amino acid sequences in *Vaccinium corymbosum* and *Arabidopsis* were performed using Clustal X (http://www.clustal.org/clustal2/) with default parameters [74]. The phylogenetic dendrogram was constructed by the neighbor-joining (NJ) method using MEGA6.0 software (https://www.megasoftware.net/) [75] with 1000 bootstrap value, and the phylogenetic tree was decorated by EvolView (https://evolgenius.info//evolview-v2/#login). In addition, the conserved motifs among VcMYB members were analyzed using MEME (v4.11.1) online program (http://meme-suite.org/index.html) [76], and the conserved motifs were confirmed according to the following parameters: the number of motifs was 10, and other parameters used the default settings. The graphics were decorated by TBtools software [77], and the phylogenetic analysis of VcMYB was the same as above. In order to analyze the sequences features of the R2 and R3 domains of VcR2R3-MYBs, the amino acid sequences of R2 and R3 repeats in *Vaccinium corymbosum* were extracted. Multiple sequence alignments of these identified R2R3-MYB proteins were aligned using Clustal X with default parameters. Multiple alignment files for R2 and R3 repeats were submitted to ggseqlogo [78] using the default settings to acquire sequence logos.

### Differential expression analysis, functional enrichment and data analysis

The data was analyzed and manipulated on the free online platform of the Majorbio Cloud Platform. To identify DEGs between two different samples, the expression level of each gene was calculated according to the FPKM. RSEM (http://deweylab.biostat.wisc.edu/rsem/) [79] was used to quantify gene abundances. R statistical package software DESeq2 (http://bioconductor.org/ packages/stats/bioc/DESeq2/) was utilized for differential expression analysis [80]. The DEGs were defined with the *p*-adjust < 0.05 and fold change ≥ 2. Veen diagrams of DEGs were generated using the Majorbio Cloud Platform. Heatmaps were generated according to the log_10_ fold-change values at MD_R/SD_R/MD_L/SD_L compared with CK_R and CK_L[81]. GO functional-annotation analysis was carried out by BLAST2GO [82] and GO(Gene Ontology, http://www.geneontology.org) database. GO functional-enrichment analysis was performed to identify which DEGs were significantly enriched in GO terms at Bonferroni-corrected *p*-value ≤ 0.05 compared with the whole- genome background. GO functional enrichment analysis was carried out by Goatools (https://github. com/tanghaibao/Goatools) [83]. GO functional-annotation, functional-enrichment and string diagram were performed using the free online platform of the Majorbio Cloud Platform. Cluster analysis of the DEGs based on the log_10_ fold-change values, was generated using Expander 7 [84] software with the K-means algorithm [85] on the Majorbio Cloud Platform. Finally, expression correlation analysis of *VcMYBs* was analyzed based on the spearman correlation algorithm with the significant differences at *q* < 0.05 and the correlation coefficient for 0.5. The expression correlation network was constructed by the free online platform of the Majorbio Cloud Platform[7]. 15 VcMYB protein sequences were used as the targets and the STRING website (https://string-db.org/cgi/input. pl) was used to predict protein-protein interactions. Their orthologs in *Arabidopsis* thaliana were appointed as references [86]. All analysis settings were set at their default values unless previously mentioned.

### Measurement of morphological and physiological indexes

The content of H_2_O_2_ and SOD activity were measured using commercial kits provided by Jiancheng Bioengineering Institute (Nanjing, China) according to the manufacturer’s instructions. According to Zhang et al, the histochemical staining of H_2_O_2_ was performed by DAB (3,3’- diaminobenzidine) method [87].

3 ~ 5 leaves below the top of the branch of seedlings were sampled and the tissue in the middle of the leaves with a width of 0.5 cm was fixed by FAA (ethanol: formaldehyde: glacial acetic acid = 90: 5:5). Paraffin section and safranine-fast green staining were performed according to the method by Miksche [88], and the slice thickness was 8 μm. The results were analyzed and photographed under a DM 3000 optical microscope [89], and leaf anatomic structures were measured and calculated using ImageJ and Nano Measurer 1.2 software. Total leaf thickness, upper epidermis thickness, palisade tissue thickness and lower epidermis thickness were observed. The CTR were calculated as follows: CTR = alisade tissue thickness/blade thickness ×100 %. Each treatment chose 3 leaves, and all experiments were carried out for three biological replicates.

### Validation of RNA-seq sequencing data by RT-qPCR

Total RNA was extracted from leaves and roots of three group seedlings using TRIzol® Reagent (OmniPlant RNA Kit(DNase I))according to the manufacturer’s instructions (Invitrogen). The first-strand cDNA was synthesized with FastQuant cDNA First-Strand Synthesis Kit (Tiangen Biotechnology, Beijing). After adjusting the cDNA template concentration, qRT-PCR assays were detected by Fluorescence Quantification PCR Kit (Tiangen Biotechnology, Beijing) on StepOnePlusTM Real-Time PCR System (ABI, USA). The primer sequences of 20 DEGs were shown in Additional file 9 and *VcUBC28* was used as the reference gene for analyzing expression data (Vashisth et al., 2011; Liang et al., 2019) [7, 90]. The RNA-seq data were displayed by log_10_ (FPKM + 1). All the qRT- PCR experiments were performed for three biological replicates.

### Statistical analysis

The IBM-SPSS version 23 was used for statistical analysis. The statistical difference analysis was performed by Dunnett’s test with *p* < 0.05 designated as significant difference.

## Supplementary Information

**Additional file 1: Table S1.** The *MYB* transcription factors in blueberry and *Arabidopsis* used in this study.

**Additional file 2: Table S2.** The detailed information of VcMYB proteins.

**Additional file 3: Fig. S1.** Validation of RNA-seq sequencing data by RT-qPCR. Expression of 16 *VcMYB* DEGs in leaf (A) or root (B) under drought stress. The left y-axis (black bars) is the relative expression level of qRT-PCR. The right y-axis (gray bars) is log_10_ (FPKM + 1) of RNA-sEq. Each value is indicated as the mean value ± standard error of three independent determinations, and different letters represent significant differences at *p* < 0.05 by Duncan’s multiple range tests.

**Additional file 4: Fig. S2.** Sequence logos of *MYB* transcription factor domains in blueberry.

**Additional file 5: Table S3.** Seventy-one highly conserved *VcR2R3-MYB* genes from blueberry.

**Additional file 6: Table S4.** Functional annotation (gene ontology) of VcMYB proteins.

**Additional file 7: Fig. S3.** Functional annotation of DEGs on the basis of gene ontology.

**Additional file 8: Table S5.** Summary of the homologous genes in blueberry and *Arabidopsis* used in this study.

**Additional file 9: Table S6.** Primer sequences were used in the experiment.

## Data Availability

All data and materials used in this study are publicly available. The datasets supporting the conclusions of this article are available in the NCBI repository (https://www.ncbi.nlm.nih.gov/bioproject/PRJNA737006/) with Bio project number PRJNA737006. The other datasets supporting the conclusions of this article are included within the article (and its additional files).

## Abbreviations

aa: Amino acid
MYB: V-myb avian myeloblastosis viral oncogene homolog
TFs: Transcription factors
DEGs: Differentially expressed genes
GO: Gene Ontology
ROS: Reactive oxygen species
RNA-Seq: RNA sequencing
SOD: Superoxide dismutase
CTR: Tissue structure tense ratio of the leaf
DAB: 3,3’-diaminobenzidine
qRT-PCR: Real-time Quantitative polymerase chain reaction
