# Supplementary material for: Genome-wide analysis of MYB transcription factors of Vaccinium corymbosum and their positive responses to drought stress
Source: BMC Genomics. 2021 Jul 22;22:565. doi: 10.1186/s12864-021-07850-5 (PMC8296672; doi:10.1186/s12864-021-07850-5)
Supplement: Supplementary file 3 — Additional file 3: Fig. S1. Validation of RNA-seq sequencing data by RT-qPCR. Expression of 16 VcMYB DEGs in leaf (A) or root (B) under drought stress. The left y-axis (black bars) is the relative expression level of qRT-PCR. The right y-axis (gray bars) is log10 (FPKM + 1) of RNA-sEq. Each value is indicated as the mean value ± standard error of three independent determinations, and different letters represent significant differences at p < 0.05 by Duncan’s multiple range tests. [file 12864_2021_7850_MOESM3_ESM.docx]

Supplementary Material

Fig. S1


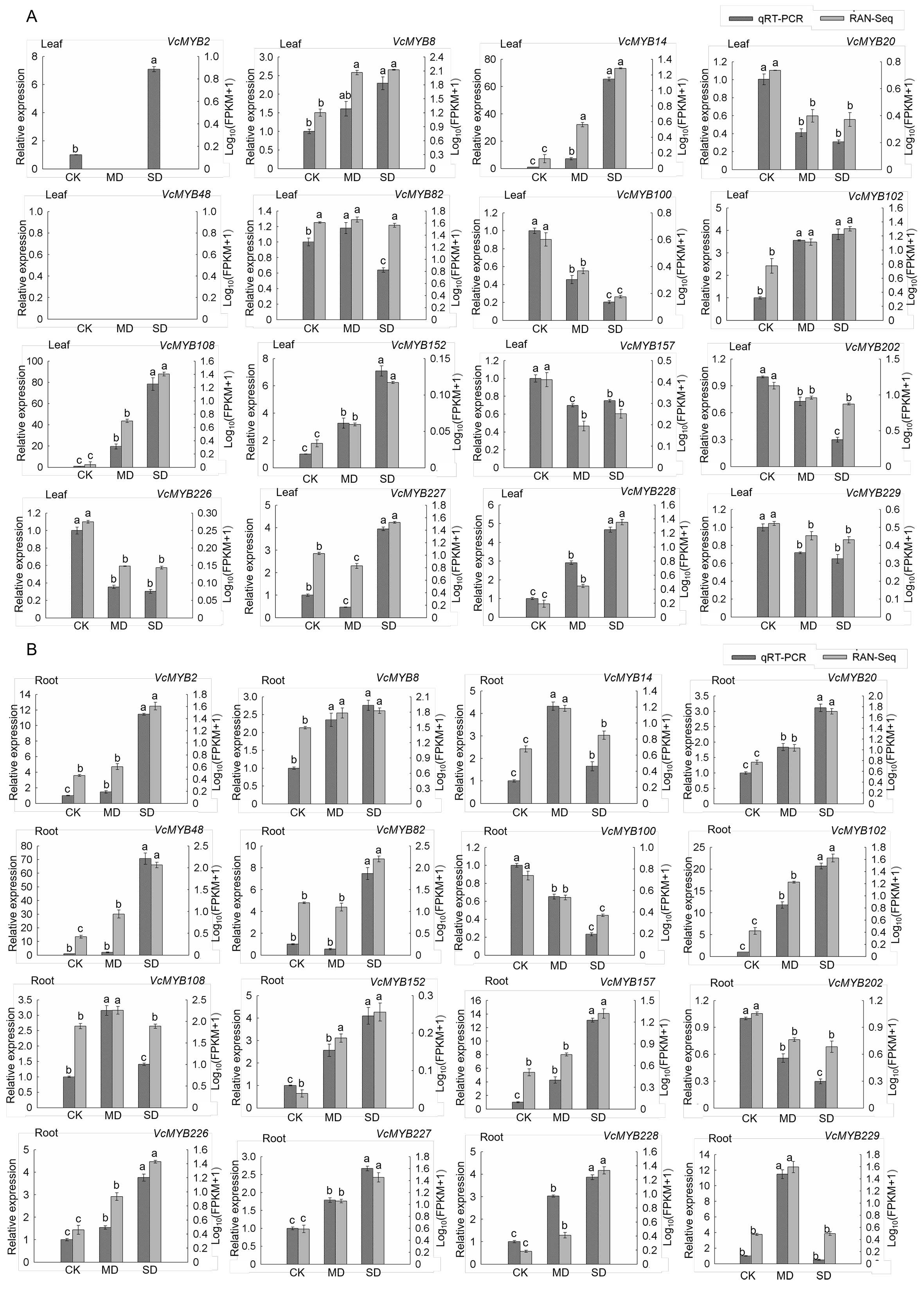


**Fig. S1.** Validation of RNA-seq sequencing data by RT-qPCR. Expression of 16 *VcMYB* DEGs in leaf (A) or root (B) under drought stress. The left y-axis (black bars) is the relative expression level of qRT-PCR. The right y-axis (gray bars) is log_10_ (FPKM + 1) of RNA-seq. Each value is indicated as the mean value ± standard error of three independent determinations, and different letters represent significant differences at *p*< 0.05 by Duncan’s multiple range test.
