# Supplementary material for: Genome-wide analysis of MYB transcription factors of Vaccinium corymbosum and their positive responses to drought stress
Source: BMC Genomics. 2021 Jul 22;22:565. doi: 10.1186/s12864-021-07850-5 (PMC8296672; doi:10.1186/s12864-021-07850-5)
Supplement: Supplementary file 4 — Additional file 4: Fig. S2. Sequence logos of MYB transcription factor domains in blueberry. [file 12864_2021_7850_MOESM4_ESM.docx]

Supplementary Material

Fig. S2

**Fig. S2.** Sequence logos of *MYB* transcription factor domains in blueberry.
