# Supplementary material for: Genome-wide analysis of MYB transcription factors of Vaccinium corymbosum and their positive responses to drought stress
Source: BMC Genomics. 2021 Jul 22;22:565. doi: 10.1186/s12864-021-07850-5 (PMC8296672; doi:10.1186/s12864-021-07850-5)
Supplement: Supplementary file 7 — Additional file 7: Fig. S3. Functional annotation of DEGs on the basis of gene ontology. [file 12864_2021_7850_MOESM7_ESM.docx]

Supplementary Material

Fig. S3

**Fig. S3.** Functional annotation of DEGs on the basis of gene ontology.
